# Neighborhood Factors, Individual Stressors, and Cardiovascular Health Among Black and White Adults in the US: The Reasons for Geographic and Racial Differences in Stroke (REGARDS) Study

**DOI:** 10.1001/jamanetworkopen.2023.36207

**Published:** 2023-09-29

**Authors:** Anika L. Hines, Michelle A. Albert, Jessica P. Blair, Deidra C. Crews, Lisa A. Cooper, D. Leann Long, April P. Carson

**Affiliations:** 1 Department of Health Behavior and Policy, Virginia Commonwealth University School of Medicine, Richmond, Virginia; 2 Department of Medicine, University of California, San Francisco, San Francisco; 3 Department of Biostatistics, University of Alabama at Birmingham, Birmingham; 4 Department of Medicine, Johns Hopkins School of Medicine, Baltimore, Maryland; 5 Department of Medicine, University of Mississippi Medical Center, Jackson

## Abstract

**Importance:**

Chronic stress has been posited to contribute to racial disparities in cardiovascular health. Investigation of whether neighborhood- and individual-level stressors mediate this disparity is needed.

**Objective:**

To examine whether racial differences in ideal cardiovascular health (ICH) are attenuated by experiences with neighborhood- and individual-level stressors within a racially and geographically diverse population sample.

**Design, Setting, and Participants:**

This cross-sectional study examined data from 7720 participants in the Reasons for Geographic and Racial Differences in Stroke (REGARDS) Study who completed the second in-home visit (2013-2016). The REGARDS study is a population-based, longitudinal study of 30 239 non-Hispanic Black and non-Hispanic White adults aged 45 years or older at baseline (2003-2007). Data for the present study were analyzed from June to July 2021 and in March 2022.

**Exposures:**

Neighborhood physical environment (eg, excessive noise, violence; scored from 7-28, with higher scores indicating more problems), neighborhood safety (scored as very safe, safe, or not safe), neighborhood social cohesion (eg, shared values; scored from 5-25, with higher scores indicating higher cohesion), perceived stress (eg, coping; scored from 0-16, with higher scores indicating greater perceived stress), and the experience of discrimination (yes or no).

**Main Outcomes and Measures:**

Ideal cardiovascular health (ICH), measured as a composite of 4 health behaviors (cigarette smoking, diet, physical activity, body mass index) and 3 health factors (blood pressure, cholesterol, and glucose levels).

**Results:**

The sample included 7720 participants (mean [SD] age, 71.9 [8.3] years; 4390 women [56.9%]; 2074 Black participants [26.9%]; and 5646 White participants [73.1%]). Black participants compared with White participants reported higher perceived stress (mean [SD] score, 3.2 [2.8] vs 2.8 [2.7]) and more often reported discrimination (77.0% vs 24.0%). Black participants also reported poorer neighborhood physical environment (mean [SD] score, 11.2 [3.8] vs 9.8 [2.9]) and social cohesion (mean [SD] score, 15.5 [2.0] vs 15.7 [1.9]) and more often reported their neighborhoods were unsafe (54.7% vs 24.3%). The odds of having a high total ICH score (ie, closer to ideal) were lower for Black adults compared with White adults, both overall (adjusted odds ratio [AOR], 0.53; 95% CI, 0.45-0.61) and by gender (men: AOR, 0.73 [95% CI, 0.57-0.93]; women: AOR, 0.45 [95% CI, 0.37-0.54]). In mediation analyses, the racial disparity in total ICH score was attenuated by neighborhood physical environment (5.14%), neighborhood safety (6.27%), neighborhood social cohesion (1.41%), and discrimination (11.01%). In stratified analyses, the factors that most attenuated the racial disparity in total ICH scores were neighborhood safety among men (12.32%) and discrimination among women (14.37%). Perceived stress did not attenuate the racial disparity in total ICH scores.

**Conclusions and Relevance:**

In this cross-sectional study of Black and White US adults aged 45 years and older, neighborhood-level factors, including safety and physical and social environments, and individual-level factors, including discrimination, attenuated racial disparities in cardiovascular health. Interventional approaches to improve ICH that separately target neighborhood context and discrimination by gender and race are warranted.

## Key Points

**Question:**

Do neighborhood factors (physical and social environments) and individual stressors (discrimination) mediate racial differences in cardiovascular health?

**Findings:**

In this cross-sectional study of 7720 participants, Black adults had poorer cardiovascular health than White adults. Neighborhood physical environment and safety attenuated racial differences in total ICH scores by 5% and 6%, respectively, while discrimination attenuated these differences by 11%.

**Meaning:**

Findings of this study suggest that racial differences in cardiovascular health are attenuated by varying experiences with neighborhood physical and social environments as well as by lived personal experiences with discrimination; approaches to improve ICH that target neighborhood factors and discrimination by gender and race are warranted.

## Teaser

This cross-sectional study examines the associations between neighborhood- and individual-level stressors and cardiovascular health among Black and White US adults aged 45 years and older.

## Introduction

In the US, Black adults have earlier onset of cardiovascular risk factors and poorer overall cardiovascular health compared with White adults.^1,2^ Psychosocial stress and stress-related coping are associated with increased cardiovascular disease (CVD) risk and may contribute to these health disparities.^3^ The weathering framework (the idea that persistent exposure to socioeconomic disadvantage, marginalization, and discrimination is associated with premature health deterioration)^4,5^ and allostatic load (dysregulated or heightened endocrine, inflammatory, and autonomic regulatory system responses resulting from chronic stress)^4^ describe how lifetime cumulative psychosocial stress (hereafter, stress) related to structural and interpersonal racism converge with health care factors to render Black Americans vulnerable to psychological and physiological responses, leading to premature, stress-related illness and mortality. Across the life span, Black people, and Black women in particular, have higher levels of allostatic load related to differential lived social experiences compared with White and male counterparts.^5^

The health equity framework for social determinants of health posits that the sociopolitical and economic context shapes social position through neighborhood environment, social and community context, and lived personal experiences of discrimination, which, ultimately, contribute to cardiovascular health inequities.^6^ For example, racial residential segregation—a manifestation of structural racism in the sociopolitical and economic context—has been associated with incident hypertension^7^ and CVD.^8^ In the Multi-Ethnic Study of Atherosclerosis,^9^ racial residential segregation was associated with cardiometabolic risk among Black participants but not among non-Hispanic White and Hispanic persons after adjusting for income. This association suggests that both individual and contextual economics are associated with CVD among Black people.^10^ A critical feature of racial residential segregation is resource disinvestment that results in neighborhood disadvantage,^11^ including concerns about crime,^12^ and fewer physical resources in the built environment, such as supermarkets^13,14^; these factors have unfavorable implications for stress and cardiovascular risk. Aspects of the neighborhood social environment, such as social cohesion or a sense of connectedness, have also been strongly associated with inflammatory stress biomarkers related to cardiovascular risk among non-Hispanic Black residents.^15^ Other contributing factors include perceptions of neighborhood safety that draw on both built and social attributes to influence health outcomes.^16^ The association between individuals’ self-reported experiences with racial discrimination and cardiovascular outcomes has been reported in the literature.^17,18^ In addition, perceived stress and discrimination have been associated with health behaviors, such as lower medication adherence, among Black patients with hypertension.^19,20^

Few studies have characterized the implications of multiple levels of simultaneous stressors for racial differences in cardiovascular health. A large cohort study of US Black adults in the South found that those experiencing higher levels of multiple stress measures were less likely to achieve ideal cardiovascular health (ICH).^21^ Ideal cardiovascular health is a composite of 4 health behaviors (cigarette smoking, diet, physical activity, body mass index) and 3 health factors (blood pressure, cholesterol and glucose levels). One study examining cumulative psychosocial stress and ICH among older female health professionals found worse ICH outcomes among Black women compared with White women.^22^ Those authors suggested that future studies should include more diverse populations, including men, participants other than health care workers, and younger participants, in addition to alternative measures of stress, including physical and social neighborhood environments.^22^ In an analysis of data from 3 cohort studies, low neighborhood social cohesion, which may elicit a chronic stress response, was also not associated with cardiovascular health.^23^ Investigating chronic stressors at both the neighborhood and individual levels may help identify which stressors are most salient for cardiovascular health and thus aid in the development of targeted interventions.

The purpose of this study was to assess whether neighborhood factors (physical environment, safety, and social cohesion) and individual stressors (perceived stress and discrimination) attenuate racial differences in ICH. We hypothesized that neighborhood factors and individual stressors would attenuate racial differences in ICH and that the observed associations would vary by gender given the well-documented disproportionate levels of stress experienced by women relative to men.^5,24^

## Methods

We analyzed cross-sectional data from the Reasons for Geographic and Racial Differences in Stroke (REGARDS) Study, a population-based, longitudinal study of 30 239 Black and White adults aged 45 years or older at baseline from 2003 to 2007.^25^ The present study used data from the REGARDS second in-home visit (2013-2016; n = 16 150 participants), which included a psychosocial questionnaire. Individuals missing data on ICH score components (n = 6603), neighborhood factors (physical environment, safety, and social cohesion; n = 1037), or individual stressors (perceived stress and discrimination; n = 790) were excluded, resulting in a final sample size of 7720 participants (Figure). Distributions of key demographics and neighborhood- and individual-level stressors were similar between persons included and excluded from the current study except for experiences with discrimination, which were reported more often by excluded individuals (91.6% of Black individuals and 74.4% of White individuals). This secondary data analysis was approved by the Virginia Commonwealth University Institutional Review Board. The REGARDS Study obtained written informed consent from all study participants and was approved by the University of Alabama at Birmingham Institutional Review Board. This study followed the Strengthening the Reporting of Observational Studies in Epidemiology (STROBE) reporting guideline.

**Figure.**
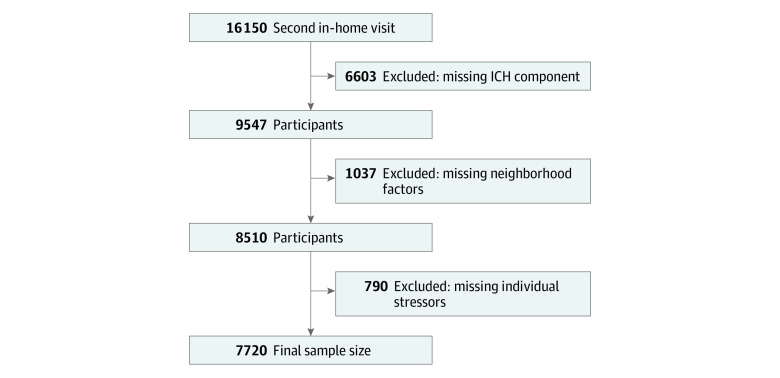
Participant Recruitment Flowchart ICH indicates ideal cardiovascular health.

### Outcomes

Ideal cardiovascular health was calculated as a composite measure of 7 components: 4 health behaviors (cigarette smoking, diet, physical activity, body mass index) and 3 health factors (blood pressure, cholesterol and glucose levels).^26^ Each component was categorized^27,28^ and given a score of 2 for ideal, 1 for intermediate, and 0 for poor (eTable 1 in Supplement 1). Component scores were summed (range, 0-14) and used to categorize overall total ICH as poor (0-4), intermediate (5-9), or ideal (≥10).

### Race

Race is a social construct. In this study, the race variable represents an indicator of shared lived experiences within sociopolitical and economic contexts in the US. Participants self-reported their race and ethnicity. Individuals who identified as non-Hispanic Black or non-Hispanic White were eligible to participate in the REGARDS study.^25^

### Neighborhood Physical Environment, Safety, and Social Cohesion

The neighborhood physical environment provides the broader structural context for individual supports (or lack thereof) that affect health, including daily stress.^12,29^ Participants rated potential problems in their neighborhood (excessive noise, heavy or speeding traffic, inadequate food shopping, lack of parks or playgrounds, trash or litter, no or poorly maintained sidewalks, violence) from 1 (not really a problem) to 4 (very serious problem)^12^ (Cronbach α = 0.73-0.83).^30^ Items were summed for a total score (range, 7-28), with a higher score indicating more problems. Participants rated their neighborhood’s safety separately on a scale of 1 (very safe) to 5 (not safe at all). Due to the small sample, these categories were collapsed to 3 levels for analytical purposes (1, very safe; 2, safe; and ≥3, unsafe).

The neighborhood social environment (ie, social cohesion) has been noted as a potential stress buffer and describes support provided through social interactions^31^ and other organizational assets. Participants rated the following statements on a scale of 1 (strongly agree) to 5 (strongly disagree): (1) people around here are willing to help their neighbors; (2) this is a close-knit neighborhood; (3) people in this neighborhood can be trusted; (4) people in this neighborhood generally do not get along; and (5) people in this neighborhood do not share the same values (Cronbach α = 0.80).^32^ Items 4 and 5 were reverse-coded; the total score was summed (range, 5-25), with higher scores indicating higher social cohesion.

### Perceived Stress and Discrimination

Participants rated their perceived stress via the 4-item Perceived Stress Scale (0, never to 4, very often): (1) feelings of controlling important things in life; (2) feeling confident about their ability to handle personal problems; (3) not coping; and (4) feelings of encountering difficulties that they could not overcome within the past month (Cronbach α = 0.74).^33^ Items 3 and 4 were reverse-coded; the total score was summed (range, 0-16), with higher scores indicating higher perceived stress.

Participants rated lifetime discrimination “due to their race, ethnicity, or color” across settings (eg, at school, getting medical care, from the police) via the Experiences of Discrimination scale, which ranges from 0 (have not experienced discrimination) to 3 (have experienced discrimination ≥4 times; Cronbach α = 0.77 to 0.81).^34^ In our analysis, we created a binary variable (did not experience discrimination [score of 0] vs experienced discrimination [score ≥1]) based on the frequency distribution of responses.

### Statistical Analysis

Demographic data included in the analysis were gender (man, woman), age (in years), highest educational attainment, annual household income, and marital status (married, divorced, widowed, separated, never married). We estimated the means for continuous variables and percentages for categorical variables and compared across racial groups using *t* tests and Cochran-Mantel-Haenszel χ^2^ tests, respectively. Due to the natural ordering of the poor, intermediate, and ideal ICH categories, we used multivariable ordinal logistic regression models to evaluate racial differences in ICH, adjusting for stressors and demographics overall and stratified by gender.^35^ Probabilities modeled were cumulated over the lower ordered values, ie, poor ICH compared with intermediate and ideal ICH. A higher odds ratio reflected a better total ICH score, or closer to ideal, compared with the poor ICH reference group.

To test our hypothesis that the racial difference in ICH was explained by neighborhood factors and individual stressors, we performed a mediation analysis with bootstrapping for each characteristic of interest: neighborhood physical environment, neighborhood safety, neighborhood social cohesion, perceived stress, and discrimination. Using the difference method approach to quantify attenuation, we estimated the association of race (acknowledging that race is a proxy for lived experiences of structural racism) with total ICH scores using estimated coefficient of race in models without and with each neighborhood factor and individual stressor separately.^36,37^ The extent to which each variable attenuated the racial differences in ICH was calculated as the ratio of the change in association compared with a reference model after adjustment for sociodemographics. Bootstrapping with a random seed and 1000 replications was used to estimate 95% CIs. Similar analyses were stratified by gender, with the extent to which each variable attenuated the racial differences in ICH calculated separately.^38^ We also estimated a model including all characteristics of interest compared with the reference model. *P* < .05 was considered to be statistically significant. Data were analyzed from June to July 2021 and in March 2022 using SAS, version 9.4 (SAS Institute, Inc).

## Results

This study included data from 7720 participants from the REGARDS study (mean [SD] age, 71.9 [8.3] years; 4390 women [56.9%] and 3330 men [43.1%]; 2074 Black participants [26.9%] and 5646 White participants [73.1%]) (Table 1). Compared with White participants, Black participants were younger and more often had a lower level of educational attainment, an annual household income less than $34 000, and were unmarried. Black participants also reported higher levels of perceived stress and discrimination and poorer neighborhood physical, social, and safety characteristics.

**Table 1.** Participant Characteristics, Overall and by Race

Characteristic	No. (%)			*P* value
	Overall (N = 7720)	Black (n = 2074)	White (n = 5646)	
Demographics				
Age, mean (SD), y	71.9 (8.3)	70.4 (8.1)	72.4 (8.3)	<.001
Gender				
Women	4390 (56.9)	1431 (69.0)	2959 (52.4)	<.001
Men	3330 (43.1)	643 (31.0)	2687 (47.6)	
Education				
Less than high school	441 (5.7)	216 (10.4)	225 (4.0)	<.001
High school graduate	1652 (21.4)	515 (24.8)	1137 (20.1)	
Some college	2031 (26.3)	611 (29.5)	1420 (25.2)	
College graduate and above	3595 (46.6)	731 (35.3)	2864 (50.7)	
Annual household income, $				
Refused to answer	1045 (13.5)	264 (12.7)	781 (13.8)	<.001
<$20 000	791 (10.3)	397 (19.1)	394 (7.0)	
20 000-34 999	1610 (20.9)	498 (24.0)	1112 (19.7)	
35 000-74 999	2514 (32.6)	651 (31.4)	1863 (33.0)	
≥75 000	1760 (22.8)	264 (12.7)	1496 (26.5)	
Marital status				
Single	359 (4.7)	176 (8.5)	183 (3.2)	<.001
Divorced	1026 (13.3)	431 (20.8)	595 (10.6)	
Married	4617 (59.9)	874 (42.2)	3743 (66.4)	
Widowed	1613 (20.9)	529 (25.5)	1084 (19.2)	
Other^a^	98 (1.3)	63 (3.0)	35 (0.6)	
Neighborhood stressors				
Neighborhood physical characteristics, mean (SD)^b^	10.2 (3.2)	11.2 (3.8)	9.8 (2.9)	<.001
Neighborhood safety				
Unsafe	2504 (32.4)	1134 (54.7)	1370 (24.3)	<.001
Safe	3075 (39.8)	683 (32.9)	2392 (42.4)	
Very safe	2141 (27.7)	257 (12.4)	1884 (33.4)	
Neighborhood social cohesion, mean (SD)^c^	15.7 (1.9)	15.5 (2.0)	15.7 (1.9)	<.001
Individual stressors				
Perceived stress, mean (SD)^d^	2.9 (2.7)	3.2 (2.8)	2.8 (2.7)	<.001
Discrimination	2949 (38.2)	1596 (77.0)	1353 (24.0)	<.001

^a^ Other indicates unknown or refused to answer.

^b^ Higher scores indicate more problems in the neighborhood.

^c^ Higher scores indicate higher social cohesion.

^d^ Higher scores indicate greater perceived stress.

Table 2 displays total and component ICH scores and categorizations (ideal, intermediate, and poor) by race and gender. Overall, Black individuals had lower mean (SD) total ICH scores than White individuals (6.7 [2.0] vs 7.7 [2.1]; *P* < .001). Compared with Black women, Black men had better mean (SD) total ICH scores (7.1 [2.0] vs 6.6 [1.9]; *P* < .001) and a higher proportion of individuals with ICH scores that were considered ideal (ie, a score of 2) (11.7% vs 7.0%; *P* < .001). White men and women had similar mean (SD) total ICH scores (7.7 [2.0] vs 7.7 [2.1]) as well as proportions of individuals with ideal ICH components (19.1% vs 21.3%), but they varied with respect to score components. For example, more White men achieved ideal physical activity than White women (32.4% vs 23.5%), but more White women achieved an ideal body mass index than White men (34.9% and 26.5%). Compared with Black men, White men had better mean (SD) total ICH scores (7.7 [2.1] vs 7.1 [2.0]) and a higher proportion with ideal ICH components (19.1% vs 11.7%) compared with Black men. Physical activity and cholesterol scores were similar between Black and White men (proportions with ideal ICH components: physical activity, 25.7% and 23.5%; cholesterol, 41.2% and 39.3%). White women had better ICH over all comparisons compared with Black women. For example, mean [SD] total ICH scores were 7.7 [2.1] for White women and 6.6 [1.9] in Black women.

**Table 2.** Ideal Cardiovascular Health (ICH) Scores and Categorizations by Race and Gender

	Overall	Black participants			*P* value, Black men vs Black women	White participants			*P* value, White men vs White women	*P* values		
		Overall	Men	Women		Overall	Men	Women		Overall Black vs White participants	Black men vs White men	Black women vs White women
ICH total score, mean (SD)	7.4 (2.1)	6.7 (2.0)	7.1 (2.0)	6.6 (1.9)	<.001	7.7 (2.1)	7.7 (2.0)	7.7 (2.1)	.55	<.001	<.001	<.001
ICH scores overall and by component, No. (%)^a^												
Overall												
Poor	629 (8.2)	278 (13.4)	68 (10.6)	210 (14.7)	<.001	351 (6.2)	159 (5.9)	192 (6.5)	.07	<.001	<.001	<.001
Intermediate	5773 (74.8)	1621 (78.2)	500 (77.8)	1121 (78.3)		4152 (73.5)	2014 (75.0)	2138 (72.3)				
Ideal	1318 (17.1)	175 (8.4)	75 (11.7)	100 (7.0)		1143 (20.2)	514 (19.1)	629 (21.3)				
Cigarette smoking												
Poor	513 (6.7)	216 (10.4)	84 (13.1)	132 (9.2)	.03	297 (5.3)	122 (4.5)	175 (5.9)	.054	<.001	<.001	<.001
Intermediate	133 (1.7)	48 (2.3)	14 (2.2)	34 (2.4)		85 (1.5)	44 (1.6)	41 (1.4)				
Ideal	7074 (91.6)	1810 (87.3)	545 (84.8)	1265 (88.4)		5264 (93.2)	2521 (93.8)	2743 (92.7)				
Physical activity												
Poor	2927 (38.3)	843 (41.1)	196 (30.9)	647 (45.7)	<.001	2084 (37.3)	833 (31.3)	1251 (42.8)	<.001	<.001	.001	.002
Intermediate	2731 (35.8)	779 (38.0)	275 (43.4)	504 (35.6)		1952 (35.0)	964 (36.3)	988 (33.8)				
Ideal	1976 (25.9)	429 (20.9)	163 (25.7)	266 (18.8)		1547 (27.7)	861 (32.4)	686 (23.5)				
Total cholesterol												
Poor	584 (7.9)	132 (6.8)	22 (3.6)	110 (8.3)	<.001	452 (8.3)	75 (2.9)	377 (13.2)	<.001	.10	.38	<.001
Intermediate	4356 (58.8)	1144 (58.9)	339 (55.2)	805 (60.6)		3212 (58.7)	1516 (57.8)	1696 (59.5)				
Ideal	2475 (33.4)	666 (34.3)	253 (41.2)	413 (31.1)		1809 (33.1)	1030 (39.3)	779 (27.3)				
Glucose												
Poor	656 (10.0)	228 (13.4)	73 (13.6)	155 (13.4)	.97	428 (8.8)	242 (10.5)	186 (7.3)	<.001	<.001	.08	<.001
Intermediate	1831 (28.0)	551 (32.4)	176 (32.7)	375 (32.3)		1280 (26.4)	725 (31.6)	555 (21.8)				
Ideal	4057 (62.0)	920 (54.2)	289 (53.7)	631 (54.4)		3137 (64.8)	1331 (57.9)	1806 (70.9)				
BMI												
Poor	2709 (35.3)	1019 (49.5)	233 (36.5)	786 (55.4)	<.001	1690 (30.0)	778 (29.0)	912 (31.0)	<.001	<.001	.001	<.001
Intermediate	2864 (37.3)	665 (32.3)	250 (39.2)	415 (29.2)		2199 (39.1)	1193 (44.5)	1006 (34.2)				
Ideal	2111 (27.5)	374 (18.2)	155 (24.3)	219 (15.4)		1737 (30.9)	709 (26.5)	1028 (34.9)				
Blood pressure												
Poor	1023 (13.3)	335 (16.2)	103 (16.1)	232 (16.2)	.15	688 (12.2)	350 (13.0)	338 (11.5)	<.001	<.001	<.001	<.001
Intermediate	5169 (67.1)	1528 (73.8)	461 (71.9)	1067 (74.6)		3641 (64.6)	1796 (66.9)	1845 (62.5)				
Ideal	1517 (19.7)	209 (10.1)	77 (12.0)	132 (9.2)		1308 (23.2)	538 (20.0)	770 (26.1)				
Diet												
Poor	5787 (75.0)	1627 (78.5)	556 (86.5)	1071 (74.8)	<.001	4160 (73.7)	2175 (91.0)	1985 (67.1)	<.001	<.001	<.001	<.001
Intermediate	1918 (24.8)	444 (21.4)	87 (13.5)	357 (25.0)		1474 (26.1)	507 (18.9)	967 (32.7)				
Ideal	15 (0.2)	3 (0.1)	0 (0)	3 (0.2)		12 (0.2)	5 (0.2)	7 (0.2)				

Abbreviation: BMI, body mass index (measured as weight in kilograms divided by height in meters squared).

^a^ ICH categories: poor, scores 0 to 4; intermediate, 5 to 9; and ideal, 10 to 14, with higher scores indicating closer to ideal.

Table 3 shows the associations between race, neighborhood factors, and individual stressors and ICH in a fully adjusted model. Black participants had lower odds of having a high total ICH score compared with their White counterparts (adjusted odds ratio [AOR], 0.53; 95% CI, 0.45-0.61). Higher neighborhood social cohesion (AOR, 1.05; 95% CI, 1.03-1.08) was associated with higher odds of having a high total ICH score. In contrast, higher perceived stress was associated with 4% lower odds (AOR, 0.96; 95% CI, 0.94-0.98) of having a high total ICH score in the fully adjusted model.

**Table 3.** Associations Among Race, Neighborhood Factors, Individual Stressors, and Ideal Cardiovascular Health (ICH)

	ICH, AOR (95% CI)^a^		
	Overall	Men	Women
Total ICH score			
Black participants	0.53 (0.45-0.61)	0.73 (0.57-0.93)	0.45 (0.37-0.54)
White participants	1 [Reference]	1 [Reference]	1 [Reference]
Demographics			
Age, y	1.00 (1.00-1.01)	1.01 (1.00-1.02)	1.00 (0.99-1.01)
Gender			
Women	1 [Reference]		
Men	0.87 (0.78-0.98)		
Education			
Less than high school	1 [Reference]	1 [Reference]	1 [Reference]
High school graduate	1.33 (1.04-1.71)	1.28 (0.84-1.97)	1.35 (0.99-1.84)
Some college	1.60 (1.24-2.05)	1.81 (1.18-2.77)	1.49 (1.09-2.03)
College graduate or above	2.49 (1.94-3.21)	2.88 (1.90-4.38)	2.32 (1.69-3.18)
Annual household income, $			
<20 000	1 [Reference]	1 [Reference]	1 [Reference]
20 000-34 999	1.16 (0.94-1.42)	0.96 (0.64-1.43)	1.22 (0.96-1.56)
35 000-74 999	1.15 (0.93-1.42)	0.90 (0.61-1.34)	1.26 (0.98-1.62)
≥75 000	1.72 (1.36-2.17)	1.14 (0.75-1.72)	2.35 (1.74-3.17)
Refused to answer	1.38 (1.10-1.74)	1.08 (0.70-1.68)	1.51 (1.15-1.98)
Marital status			
Single	1 [Reference]	1 [Reference]	1 [Reference]
Married	1.42 (1.09-1.84)	1.08 (0.69-1.70)	1.56 (1.13-2.16)
Divorced	1.44 (1.08-1.90)	0.83 (0.49-1.41)	1.78 (1.27-2.49)
Widowed	1.13 (0.86-1.49)	0.90 (0.54-1.51)	1.28 (0.92-1.77)
Other^b^	1.21 (0.72-2.03)	0.77 (0.31-1.89)	1.53 (0.81-2.90)
Neighborhood stressors			
Neighborhood physical characteristics	0.98 (0.96-0.99)	0.97 (0.94-1.00)	0.98 (0.96-1.00)
Neighborhood safety			
Unsafe	1 [Reference]	1 [Reference]	1 [Reference]
Safe	0.95 (0.84-1.09)	1.03 (0.83-1.27)	0.90 (0.76-1.07)
Very safe	0.94 (0.81-1.10)	1.02 (0.81-1.30)	0.88 (0.72-1.07)
Neighborhood social cohesion	1.05 (1.03-1.08)	1.04 (1.00-1.09)	1.07 (1.03-1.11)
Individual stressors			
Perceived stress	0.96 (0.94-0.98)	0.97 (0.94-1.01)	0.95 (0.92-0.97)
Discrimination	0.89 (0.79-1.01)	1.01 (0.83-1.23)	0.80 (0.68-0.95)

Abbreviation: AOR, adjusted odds ratio.

^a^ Models are fully adjusted for demographic characteristics, neighborhood physical environment, neighborhood safety, neighborhood social cohesion, perceived stress, and discrimination.

^b^ Other indicates unknown or refused to answer.

In analyses stratified by gender (Table 3), Black men had 27% lower odds of having a high total ICH score (AOR, 0.73; 95% CI, 0.57-0.93) compared with White men in the fully adjusted model. Among women, Black women had 55% lower odds of having a higher total ICH score (AOR, 0.45; 95% CI, 0.37-0.54) compared with White women in the fully adjusted model. Higher educational level was associated with ICH. For example, compared with having less than a high school education, having a college education was associated with higher total ICH score for men (AOR, 2.88; 95% CI, 1.90-4.38) and women (AOR 2.32; 95% CI, 1.69-3.18). Compared with being single, being married (AOR, 1.56; 95% CI, 1.13-2.16) or divorced (AOR, 1.78; 95% CI, 1.27-2.49) was associated with higher odds of having a higher total ICH score in women. Social cohesion was associated with higher odds of having a higher total ICH score (AOR, 1.07; 95% CI, 1.03-1.11) among women. Perceived stress (AOR, 0.95; 95% CI, 0.92-0.97) and discrimination (AOR, 0.80; 95% CI, 0.68-0.95) were associated with lower odds of having a higher total ICH score among women. Stepwise models adding characteristics individually are provided in eTables 2 to 4 in Supplement 1.

The neighborhood- and individual-level factors that attenuated the racial differences in ICH scores among Black and White participants are presented in Table 4. In the reference model that was adjusted for sociodemographic variables, Black participants had lower total ICH scores overall (β = −0.70; 95% CI, −0.81 to −0.59), among men (β = −0.36; 95% CI, −0.53 to −0.18), and among women (β = −0.88; 95% CI, −1.01 to −0.74) compared with White participants. The overall difference in total ICH scores between Black and White participants was attenuated by neighborhood physical environment (5.14%; 95% CI, 3.27%-7.15%), neighborhood safety (6.27%; 95% CI, 2.46%-10.05%), social cohesion (1.41%; 95% CI, 0.48%-2.60%), and discrimination (11.01%; 95% CI, 3.22%-19.15%). Among men, the racial difference in total ICH scores was attenuated by neighborhood physical environment (5.59%; 95% CI, 1.51%-10.23%), neighborhood safety (12.32%; 95% CI, 2.08%-22.42%), and neighborhood social cohesion (4.95%; 95% CI, 1.24%-9.26%). Among women, the racial difference in total ICH scores was attenuated by neighborhood physical environment (4.81%; 95% CI, 2.27%-7.32%), neighborhood safety (4.38%; 95%, 0.33%-8.98%), and discrimination (14.37%; 95% CI, 6.50%-22.23%). In the model including all characteristics of interest, the racial difference in total ICH scores was attenuated overall (12.27%; 95% CI, 3.58%-20.86%) and among women (13.68%; 95% CI, 4.51%-23.01%) but not among men (9.15; 95% CI, −17.60% to 36.67%).

**Table 4.** Attenuation of Racial Differences in Ideal Cardiovascular Health by Neighborhood Factors and Individual Stressors Overall and Stratified by Gender

Model	% Change in β (95% CIs)^a^^,^^b^^,^^c^		
	Study population	Men	Women
Model 1: adjusted for demographics^d^	1 [Reference]	1 [Reference]	1 [Reference]
Model 2: adjusted for model 1 + neighborhood physical environment	5.14 (3.27 to 7.15)	5.59 (1.51 to 10.23)	4.81 (2.27 to 7.32)
Model 3: adjusted for model 1 + neighborhood safety	6.27 (2.46 to 10.05)	12.32 (2.08 to 22.42)	4.38 (0.33 to 8.98)
Model 4: adjusted for model 1 + neighborhood social cohesion	1.41 (0.48 to 2.60)	4.95 (1.24 to 9.26)	0.76 (−0.25 to 1.93)
Model 5: adjusted for model 1 + perceived stress	1.18 (−0.07 to 2.64)	1.59 (−0.94 to 4.94)	1.22 (−0.57 to 2.90)
Model 6: adjusted for model 1 + discrimination	11.01 (3.22 to 19.15)	3.62 (−21.8 to 29.32)	14.37 (6.50 to 22.23)
Model 7: adjusted for model 1 + all potential mediators (fully adjusted)	12.27 (3.58 to 20.86)	9.15 (−17.60 to 35.67)	13.68 (4.51 to 23.01)

^a^ Ideal cardiovascular health is treated as a continuous variable.

^b^ Calculated as the difference in the β coefficient for race in the reference model (adjusted for age, education, income, and marital status) and separate models further adjusted for each characteristic of interest compared with the β coefficient for race in the reference model.

^c^ Bootstrapped CIs.

^d^ The β coefficients for race in the reference model (Black participants compared with White participants) are: overall, β = −0.70 (95% CI, −0.81 to −0.59); men, β = −0.36 (95% CI, −0.53 to −0.18); and women, β = −0.88 (95% CI, −1.01 to −0.74).

## Discussion

This study sought to characterize the role of neighborhood-level factors and individual-level stressors in racial differences in ICH among Black and White individuals. We found that Black participants had a higher prevalence of stressors, such as stress-related neighborhood physical environment, neighborhood safety, perceived stress, and discrimination, and lower levels of neighborhood social cohesion.^32^ Consistent with our hypothesis grounded in the social determinants of health framework^6^—that disproportionate exposures to stressors underlie racial differences in cardiovascular health—neighborhood physical environment, safety, and social cohesion and individual experiences with racial discrimination (and not perceived stress, which is a noncontextual measure of general stress) partially mediated the association between race and ICH, together attenuating approximately 12% of the observed racial difference in the fully adjusted model. Moreover, the proportions of the racial differences mediated by specific stressors, such as neighborhood safety and discrimination, ranged from approximately 1% to 14% and varied by gender.

Previous studies have indicated that women have higher rates of ICH than men^39,40,41^; however, this study offers further nuance suggesting gender differences by race. Within race groups, we found no difference in ICH between White men and White women, while Black men had better ICH compared with Black women. Further investigation is warranted to understand these unexpected gender associations. Racial differences in ICH persisted in within-gender comparisons. Whereas Black men had 27% lower odds of having a higher total ICH score than White men, Black women had 55% lower odds than White women—more than double the racial gap among men. A previous study found a similar racial disparity among female health professionals after adjusting for cumulative stress.^22^ The current analysis also offers new insight regarding ICH disparities among men, who typically have a higher incidence of cardiovascular disease but have better anticipated outcomes than women after acute cardiovascular events.^42^

Demographic correlates of ICH also differed by gender, illuminating psychosocial implications. Education level was associated with ICH in a seemingly dose-dependent association, such that the odds of having a higher total ICH score increased with each ordinal level of educational attainment. Educational attainment is strongly associated with health outcomes among White individuals.^43^ Education may be a more salient factor in ICH among Black people and women,^44^ who reap diminishing returns—earning less than White men at every education level^45^—and subsequently, experience poorer health care,^46^ subjective health,^47^ and cardiovascular outcomes.^48^ Black women, specifically, earn less than men and White women,^49^ which may be reflected in the ICH outcomes in this study. We found that higher income was related to ICH among women but not men in this study. In another example, marital status, frequently operationalized as a proxy of social support, was not associated with ICH among men despite previous evidence of marriage’s protection against poor health^50^ and cardiovascular outcomes^51^ in this group. On the other hand, our finding that being married or divorced was associated with higher total ICH scores among women is consistent with previous work suggesting some economic benefits (especially among White women) and, consequently, health benefits for women related to marriage vs being single^52^ (even if the marriage has dissolved). In this way, social support may be operationalized differently within the context of healthy cardiovascular aging among women and men. It should be further noted that benefits of marriage may not be conferred uniformly across racial groups, in that Black women are less likely to marry at all, marry later in life, and have higher rates of marital instability compared with White and Hispanic women.^53^

Among men, the racial difference in ICH was partially explained by neighborhood physical environment (an inventory of problematic disorder, resources, and violence), safety, and social cohesion (sentiments of community trust and shared values). Neighborhood disorder has been associated with crime and violence, which may collectively influence mental health outcomes.^12,29^ A growing body of literature indicates that exposure to violence (reflected in our neighborhood measure) may be associated with hypertension and other cardiovascular outcomes.^54^ Over the life span, men represent a greater proportion of perpetrators and victims of violent crime compared with women, although gender differences in violence appear to be decreasing over time^55^ and are reduced at higher levels of disadvantage.^56^ Despite these slowly shifting trends, community violence presents a more relevant threat and, thus, a more prominent stressor among men relative to women. Moreover, neighborhood disadvantage increases the exposure to peer violence for both men and women.^57^ For Black individuals, whose communities are policed more often,^58^ threats of fatal police encounters, which occur at rates nearly 3 to 5 times that of their White counterparts,^59,60^ serve as an additional type of potential violent stressor. Indeed, studies examining the outcomes of the killing of unarmed Black people related to neighborhood policing have found adverse mental health implications for Black communities.^61,62,63^ Additionally, circumstances of heightened police presence may spark vigilant anticipatory coping (a strategy used to prepare for possible mistreatment related to race^64^), which is associated with increased hypertension and obesity risk among Black individuals^65,66^ and large arterial elasticity among boys.^67^ Vigilant coping against racial discrimination may help to buffer against depression^68^ but exacerbate hypertension risk^69^ among Black individuals. In this study, discrimination was not associated with racial differences in ICH among men. Indeed, it is possible that the disproportionate threat of physical harm to Black men could be perceived as a form of discrimination, thereby subsuming the discrimination construct as measured. More research is needed to understand the implications of violence and neighborhood policing as a neighborhood stressor for cardiovascular outcomes. Additionally, neighborhood disadvantage is associated with diminished access to healthy food options and inability to safely perform physical activity.^12,13,14^ Of note, social cohesion helped to explain racial differences in ICH among men only. This finding suggests that neighborhood social conditions constituting a more communal level of social support rather than solely that of the familial level may help to counter some of the stress faced by Black men, with implications for improved ICH.

Discrimination explained 14.37% of the racial disparity in ICH among women only. Experiences with discrimination have been associated with increased cardiovascular risk factors, such as obesity and hypertension.^70,71^ Discrimination may be particularly detrimental to the health outcomes of Black women due to social disadvantage in both race and gender domains.^72^ Experiencing racial discrimination may also reduce the effectiveness of interventions to improve cardiometabolic health within this group.^73^ Black women have higher levels of chronic stress over the life span as measured by allostatic load, which is likely related to the disadvantages of this dual identity.^5,24^ One category of unique discrimination experiences by Black women—gendered racial microaggressions^74^—has been associated with poorer mental health and self-reported health. Additional studies are required to understand the role of these unique discrimination experiences in cardiovascular health. Moreover, it is unclear how common coping mechanisms, such as the superwoman schema (a frame describing the associations between stress and health among African American women),^75^ impact cardiovascular outcomes, although possible pathways have been described.^76^ Taken together, understanding racial and gender differences in ICH should be considered intersectionally through the “interconnected nature of social categorizations, such as race, class, and gender,” which may “create overlapping and interdependent systems of discrimination or disadvantage.”^77^

### Strengths and Limitations

We believe that this study offers new insight regarding the role of varying stressors on racial and gender disparities in ICH. A study strength is the large, population-based cohort of Black and White individuals with rigorously assessed physiological measures and validated measures of neighborhood attributes and individual stressors, including discrimination.

Nonetheless, the findings of this study should be interpreted within the context of the following limitations. First, this study is cross-sectional and thus causation cannot be determined. Second, because a higher proportion of individuals excluded from the analysis experienced discrimination regardless of race, the association between discrimination and ICH warrants further investigation. Third, we discuss stressors in terms of neighborhood factors and individual stressors (ie, lived personal experience) to convey interpretable opportunities for intervention through clinical and population health strategies with the understanding that these constructs are interrelated. Finally, the ICH construct does not capture health in totality. Despite these limitations, evaluating the differential implications of stressors for cardiovascular health by race is important given that CVD is the greatest contributor to racial disparities in premature mortality in the US.^78^

## Conclusions

In this cross-sectional study that used a population-based sample of middle-aged and older adults, Black adults had poorer ICH than their White counterparts, with larger racial differences noted among women than men. Neighborhood factors helped to attenuate racial disparities in ICH, especially among men, while discrimination attenuated differences among women only. Further work is needed to explore these associations in other diverse populations, particularly differential associations between discrimination and ICH by gender. From a public health standpoint, these data suggest that interventional approaches that separately target neighborhood factors and discrimination by gender and race are warranted. For example, addressing unique stressors that tie into the superwoman schema could be more consequential in Black women, whereas addressing the impact of neighborhood violence may be differentially more important for Black men.

## Notes

Supplement 1. **eTable 1.** Ideal Cardiovascular Health Domains as Implemented in REGARDS, Including Criteria and Scoring **eTable 2.** Association Among Race, Individual and Community Stressors, and Ideal Cardiovascular Health Overall **eTable 3.** Association Among Race, Individual and Community Stressors, and Ideal Cardiovascular Health for Men **eTable 4.** Association Among Race, Individual and Community Stressors, and Ideal Cardiovascular Health for Women

Supplement 2.
Data Sharing Statement
