# Supplementary material for: Neighborhood Factors, Individual Stressors, and Cardiovascular Health Among Black and White Adults in the US: The Reasons for Geographic and Racial Differences in Stroke (REGARDS) Study
Source: JAMA Netw Open. 2023 Sep 29;6(9):e2336207. doi: 10.1001/jamanetworkopen.2023.36207 (PMC10543067; doi:10.1001/jamanetworkopen.2023.36207)
Supplement: Supplement 1. — eTable 1. Ideal Cardiovascular Health Domains as Implemented in REGARDS, Including Criteria and Scoring eTable 2. Association Among Race, Individual and Community Stressors, and Ideal Cardiovascular Health Overall eTable 3. Association Among Race, Individual and Community Stressors, and Ideal Cardiovascular Health for Men eTable 4. Association Among Race, Individual and Community Stressors, and Ideal Cardiovascular Health for Women [file jamanetwopen-e2336207-s001.pdf]

Supplemental Online Content

Hines AL, Albert MA, Blair JP, et al. Neighborhood factors, individual stressors, and cardiovascular health among Black and White adults in the US: the Reasons for Geographic and Racial Differences in Stroke (REGARDS) Study. *JAMA Netw Open*. 2023;6(9):e2336207. doi:10.1001/jamanetworkopen.2023.36207

eTable 1. Ideal Cardiovascular Health Domains as Implemented in REGARDS, Including Criteria and Scoring

eTable 2. Association Among Race, Individual and Community Stressors, and Ideal Cardiovascular Health Overall

eTable 3. Association Among Race, Individual and Community Stressors, and Ideal Cardiovascular Health for Men

eTable 4. Association Among Race, Individual and Community Stressors, and Ideal Cardiovascular Health for Women

This supplemental material has been provided by the authors to give readers additional information about their work.

eTable 1. Ideal Cardiovascular Health Domains as Implemented in REGARDS, Including Criteria and Scoring

Ideal Cardiovascular Health Domains	Implemented in REGARDS	Criteria	Score
Diet	Number of criteria met from Block Food Frequency (98)<br>• ≥4.5 cups/day of fruits and vegetables<br>• 200 g/wk of fish<br>• >0.1 ratio of fiber to carbohydrates per day,<br>• ≤450 calories/day of sweets/desserts and<br>• <1500 mg/d of sodium	4 to 5<br>2 to 3<br>0 to 1	2<br>1<br>0
Physical activity	Times per week engage in intense physical activity, enough to work up a sweat	≥4<br>1-3<br>0	2<br>1<br>0
Smoking	Current smoking status	Never use or quit > 12 months ago<br>Former smoker quit ≤12 months ago<br>Current smoker	2<br>1<br>0
BMI	BMI (kg/m^2)	<25.0<br>25.0 – 29.9<br>>30.0	2<br>1<br>0
Total cholesterol	Total cholesterol (mg/dL)	<200<br>200-239 or treated to goal<br>≥240	2<br>1<br>0
Fasting glucose	Fasting glucose (mg/dL)	Fasting <100 or non-fasting <140<br>Fasting 100-125, non-fasting 140-199, or treated to goal<br>Fasting ≥126 or non-fasting ≥200	2<br>1<br>0
Blood pressure	Systolic and diastolic blood pressure (mmHg)	<120/<80<br>SBP 120-139 or DBP 80-89, or treated to goal<br>≥140/≥90	2<br>1<br>0

**eTable 2. Association Among Race, Individual and Community Stressors, and Ideal Cardiovascular Health Overall**							
	Model 1 Race only (unadjusted)	*Model 2 Model 1 + demographics*	*Model 3 Model 2 + physical community environment*	*Model 4 Model 3 + community safety*	*Model 5 Model 4 + community social environment*	*Model 6 Model 5 + perceived stress*	*Model 7 Model 6 + perceived discrimination (Fully adjusted)*
	**OR 95% CI**	**OR 95% CI**	**OR 95% CI**	**OR 95% CI**	**OR 95% CI**	**OR 95% CI**	**OR 95% CI**
**Black versus White (ref).**	**0.39 (0.35, 0.45)**	**0.49 (0.43, 0.55)**	**0.50 (0.44, 0.57)**	**0.50 (0.44, 0.57)**	**0.50 (0.44, 0.57)**	**0.50 (0.44, 0.57)**	**0.53 (0.45, 0.61)**
Demographics							
Age (years)		1.01 (1.00, 1.01)	1.01 (1.00, 1.01)	1.01 (1.00, 1.01)	1.00 (1.00, 1.01)	1.00 (1.00, 1.01)	1.00 (1.00, 1.01)
Gender							
Women		Ref.	Ref.	Ref.	Ref.	Ref.	Ref.
Men		**0.88 (0.78, 0.98)**	**0.88 (0.78, 0.98)**	**0.88 (0.78, 0.98)**	**0.88 (0.79, 0.99)**	**0.87 (0.78, 0.97)**	**0.87 (0.78, 0.98)**
Education							
Less than high school		Ref.	Ref.	Ref.	Ref.	Ref.	Ref.
High school graduate		**1.37 (1.07, 1.76)**	**1.35 (1.05, 1.73)**	**1.35 (1.05, 1.73)**	**1.35 (1.05, 1.73)**	**1.33 (1.03, 1.70)**	**1.33 (1.04, 1.71)**
Some college		**1.63 (1.27, 2.09)**	**1.60 (1.25, 2.05)**	**1.60 (1.25, 2.05)**	**1.61 (1.26, 2.07)**	**1.57 (1.23, 2.02)**	**1.60 (1.24, 2.05)**
College graduate or above		**2.57 (2.00, 3.29)**	**2.50 (1.95, 3.20)**	**2.50 (1.95, 3.20)**	**2.53 (1.97, 3.25)**	**2.45 (1.91, 3.15)**	**2.49 (1.94, 3.21)**
Household Income							
< $20k		Ref.	Ref.	Ref.	Ref.	Ref.	Ref.
$20k - $34k		1.19 (0.97, 1.46)	1.17 (0.95, 1.43)	1.17 (0.95, 1.43)	1.17 (0.95, 1.44)	1.15 (0.93, 1.41)	1.16 (0.94, 1.42)
$35k - $74k		1.23 (1.00, 1.51)	1.18 (0.95, 1.45)	1.18 (0.95, 1.45)	1.19 (0.96, 1.46)	1.15 (0.93, 1.41)	1.15 (0.93, 1.42)
≥ $75k		**1.88 (1.49, 2.37)**	**1.78 (1.41, 2.24)**	**1.77 (1.40, 2.24)**	**1.80 (1.42, 2.27)**	**1.71 (1.35, 2.17)**	**1.72 (1.36, 2.17)**
Refused		**1.44 (1.15, 1.81)**	**1.39 (1.11, 1.75)**	**1.39 (1.11, 1.75)**	**1.41 (1.12, 1.78)**	**1.37 (1.09, 1.73)**	**1.38 (1.10, 1.74)**
Marital Status							
Single		Ref.	Ref.	Ref.	Ref.	Ref.	Ref.
Married		**1.47 (1.14, 1.91)**	**1.44 (1.11, 1.87)**	**1.44 (1.11, 1.87)**	**1.41 (1.09, 1.83)**	**1.42 (1.09, 1.84)**	**1.42 (1.09, 1.84)**
Divorced		**1.46 (1.10, 1.93)**	**1.42 (1.07, 1.89)**	**1.42 (1.07, 1.89)**	**1.43 (1.08, 1.90)**	**1.43 (1.08, 1.89)**	**1.44 (1.08, 1.90)**
Widowed		1.17 (0.89, 1.55)	1.14 (0.87, 1.51)	1.14 (0.87, 1.51)	1.13 (0.86, 1.49)	1.13 (0.86, 1.50)	1.13 (0.86, 0.49)
Other		1.19 (0.71, 2.01)	1.17 (0.69, 1.96)	1.17 (0.69, 1.97)	1.16 (0.69, 1.96)	1.21 (0.72, 2.03)	1.21 (0.72, 2.03)
Neighborhood Stressors							

Neighborhood physical characteristics			**0.97 (0.95, 0.99)**	**0.97 (0.95, 0.99)**	**0.97 (0.95, 0.99)**	**0.97 (0.96, 0.99)**	**0.98 (0.96, 0.99)**
Neighborhood safety							
Unsafe				Ref.	Ref.	Ref.	Ref.
Safe				1.00 (0.87, 1.14)	0.97 (0.85, 1.11)	0.96 (0.84, 1.10)	0.95 (0.84, 1.09)
Very safe				1.01 (0.87, 1.17)	0.97 (0.83, 1.13)	0.95 (0.81, 1.10)	0.94 (0.81, 1.10)
Neighborhood social cohesion					**1.05 (1.03, 1.08)**	**1.06 (1.03, 1.09)**	**1.05 (1.03, 1.08)**
Individual Stressors							
Perceived Stress						**0.96 (0.94, 0.98)**	**0.96 (0.94, 0.98)**
Discrimination							0.89 (0.79, 1.01)

eTable 3. Association Among Race, Individual and Community Stressors, and Ideal Cardiovascular Health for Men

	Model 1 Race only (unadjusted)	*Model 2 Model 1 + demographics*	*Model 3 Model 2 + physical community environment*	*Model 4 Model 3 + community safety*	*Model 5 Model 4 + community social environment*	*Model 6 Model 5 + perceived stress*	*Model 7 Model 6 + perceived discrimination (Fully adjusted)*
	**OR 95% CI**	**OR 95% CI**	**OR 95% CI**	**OR 95% CI**	**OR 95% CI**	**OR 95% CI**	**OR 95% CI**
**Black versus White (ref).**	**0.55 (0.44, 0.67)**	**0.71 (0.57, 0.88)**	**0.72 (0.58, 0.90)**	**0.73 (0.58, 0.91)**	**0.73 (0.59, 0.91)**	**0.73 (0.59, 0.91)**	**0.73 (0.57, 0.93)**
Demographics							
Age (years)		1.01 (1.00, 1.02)	1.01 (1.00, 1.02)	1.01 (1.00, 1.02)	1.01 (1.00, 1.02)	1.01 (1.00, 1.02)	1.01 (1.00, 1.02)
Education							
Less than high school		Ref.	Ref.	Ref.	Ref.	Ref.	Ref.
High school graduate		1.32 (0.86, 2.02)	1.30 (0.85, 2.00)	1.30 (0.85, 1.99)	1.29 (0.84, 1.97)	1.28 (0.84, 1.97)	1.28 (0.84, 1.97)
Some college		**1.87 (1.22, 2.86)**	**1.83 (1.20, 2.80)**	**1.82 (1.19, 2.79)**	**1.82 (1.19, 2.79)**	**1.81 (1.18, 2.77)**	**1.81 (1.18, 2.77)**
College graduate or above		**3.00 (1.98, 4.55)**	**2.92 (1.93, 4.44)**	**2.90 (1.91, 4.41)**	**2.92 (1.92, 4.44)**	**2.89 (1.90, 4.38)**	**2.88 (1.90, 4.38)**
Household Income							
< $20k		Ref.	Ref.	Ref.	Ref.	Ref.	Ref.
$20k - $34k		1.00 (0.67, 1.49)	0.97 (0.65, 1.45)	0.97 (0.65, 1.45)	0.98 (0.65, 1.46)	0.96 (0.64, 1.43)	0.96 (0.64, 1.43)
$35k - $74k		0.97 (0.65, 1.43)	0.93 (0.63, 1.37)	0.93 (0.63, 1.37)	0.93 (0.63, 1.38)	0.91 (0.61, 1.34)	0.90 (0.61, 1.34)
≥ $75k		1.25 (0.83, 1.88)	1.18 (0.78, 1.78)	1.17 (0.78, 1.77)	1.18 (0.78, 1.78)	1.14 (0.75, 1.72)	1.14 (0.75, 1.72)
Refused		1.16 (0.75, 1.79)	1.10 (0.78, 1.78)	1.10 (0.71, 1.71)	1.11 (0.72, 1.73)	1.08 (0.70, 1.68)	1.08 (0.70, 1.68)
Marital Status							
Single		Ref.	Ref.	Ref.	Ref.	Ref.	Ref.
Married		1.17 (0.75, 1.84)	1.12 (0.71, 1.76)	1.20 (0.71, 1.76)	1.10 (0.70, 1.72)	1.08 (0.69, 1.70)	1.08 (0.69, 1.70)
Divorced		0.88 (0.52, 1.49)	0.85 (0.50, 1.44)	0.85 (0.50, 1.44)	0.84 (0.50, 1.43)	0.83 (0.49, 1.41)	0.83 (0.49, 1.41)
Widowed		0.97 (0.58, 1.62)	0.93 (0.55, 1.55)	0.92 (0.55, 1.54)	0.91 (0.55, 1.53)	0.90 (0.54, 1.51)	0.90 (0.54, 1.51)
Other		0.82 (0.34, 2.02)	0.79 (0.32, 1.94)	0.79 (0.32, 1.94)	0.76 (0.31, 1.89)	0.77 (0.31, 1.90)	0.77 (0.31, 1.89)
Neighborhood Stressors							
Neighborhood physical characteristics			**0.97 (0.94, 0.99)**	0.97 (0.94, 1.00)	0.97 (0.94, 1.00)	0.97 (0.94, 1.00)	0.97 (0.94, 1.00)
Neighborhood safety							
Unsafe				Ref.	Ref.	Ref.	Ref.

Safe				1.05 (0.85, 1.30)	1.03 (0.84, 1.28)	1.02 (0.83, 1.27)	1.03 (0.83, 1.27)
Very safe				1.07 (0.84, 1.35)	1.04 (0.82, 1.32)	1.02 (0.81, 1.30)	1.02 (0.81, 1.30)
Neighborhood social cohesion					1.04 (1.00, 1.09)	1.04 (1.00, 1.09)	1.04 (1.00, 1.09)
Individual Stressors							
Perceived Stress						0.98 (0.94, 1.01)	0.97 (0.94, 1.01)
Discrimination							1.01 (0.83, 1.23)

eTable 4. Association Among Race, Individual and Community Stressors, and Ideal Cardiovascular Health for Women							
	Model 1 Race only (unadjusted)	*Model 2 Model 1 + demographics*	*Model 3 Model 2 + physical community environment*	*Model 4 Model 3 + community safety*	*Model 5 Model 4 + community social environment*	*Model 6 Model 5 + perceived stress*	*Model 7 Model 6 + perceived discrimination (Fully adjusted)*
	**OR 95% CI**	**OR 95% CI**	**OR 95% CI**	**OR 95% CI**	**OR 95% CI**	**OR 95% CI**	**OR 95% CI**
**Black versus White (ref).**	**0.34 (0.29, 0.40)**	**0.40 (0.34, 0.47)**	**0.42 (0.35, 0.49)**	**0.41 (0.35, 0.49)**	**0.41 (0.34, 0.48)**	**0.40 (0.34, 0.48)**	**0.45 (0.37, 0.54)**
Demographics							
Age (years)		1.01 (1.00, 1.02)	1.01 (1.00, 1.01)	1.01 (1.00, 1.01)	1.01 (1.00, 1.01)	1.01 (1.00, 1.01)	1.00 (0.99, 1.01)
Education							
Less than high school		Ref.	Ref.	Ref.	Ref.	Ref.	Ref.
High school graduate		**1.39 (1.02, 1.89)**	1.36 (1.00, 1.86)	1.37 (1.00, 1.86)	**1.38 (1.01, 1.87)**	1.33 (0.97, 1.81)	1.35 (0.99, 1.84)
Some college		**1.50 (1.10, 2.04)**	**1.47 (1.08, 2.00)**	**1.48 (1.08, 2.01)**	**1.50 (1.10, 2.04)**	**1.43 (1.05, 1.95)**	**1.49 (1.09, 2.03)**
College graduate or above		**2.35 (1.72, 3.21)**	**2.29 (1.67, 3.13)**	**2.29 (1.68, 3.14)**	**2.34 (1.71, 3.20)**	**2.22 (1.62, 3.04)**	**2.32 (1.69, 3.18)**
Household Income							
< $20k		Ref.	Ref.	Ref.	Ref.	Ref.	Ref.
$20k - $34k		1.24 (0.97, 1.58)	1.22 (0.96, 1.56)	1.22 (0.96, 1.56)	1.23 (0.97, 1.57)	1.21 (0.95, 1.55)	1.22 (0.96, 1.56)
$35k - $74k		**1.33 (1.04, 1.71)**	1.28 (1.00, 1.65)	1.28 (1.00, 1.65)	**1.31 (1.01, 1.68)**	1.25 (0.97, 1.61)	1.26 (0.98, 1.62)
≥ $75k		**2.55 (1.90, 3.42)**	**2.41 (1.79, 3.25)**	**2.42 (1.80, 3.27)**	**2.49 (1.84, 3.35)**	**2.34 (1.73, 3.16)**	**2.35 (1.74, 3.17)**
Refused		**1.55 (1.18, 2.03)**	**1.51 (1.15, 1.98)**	**1.51 (1.15, 1.98)**	**1.54 (1.17, 2.02)**	**1.49 (1.14, 1.96)**	**1.51 (1.15, 1.98)**
Marital Status							
Single		Ref.	Ref.	Ref.	Ref.	Ref.	Ref.
Married		**1.57 (1.14, 2.16)**	**1.55 (1.12, 2.13)**	**1.55 (1.13, 2.14)**	**1.52 (1.10, 2.09)**	**1.57 (1.14, 2.16)**	**1.56 (1.13, 2.16)**
Divorced		**1.75 (1.25, 2.44)**	**1.72 (1.23, 2.41)**	**1.72 (1.23, 2.40)**	**1.74 (1.24, 2.43)**	**1.76 (1.26, 2.46)**	**1.78 (1.27, 2.49)**
Widowed		1.30 (0.93, 1.80)	1.28 (0.92, 1.77)	1.28 (0.92, 1.78)	1.26 (0.91, 1.76)	1.28 (0.92, 1.79)	1.28 (0.92, 1.77)
Other		1.43 (0.76, 2.72)	1.42 (0.75, 2.69)	1.42 (0.75, 2.69)	1.45 (0.76, 2.74)	1.54 (0.81, 2.92)	1.53 (0.81, 2.90)
Neighborhood Stressors							
Neighborhood physical characteristics			**0.97 (0.95, 0.99)**	**0.97 (0.95, 0.99)**	0.97 (0.95, 1.00)	0.98 (0.95, 1.00)	0.98 (0.96, 1.00)
Neighborhood safety							
Unsafe				Ref.	Ref.	Ref.	Ref.

Safe				0.96 (0.81, 1.14)	0.93 (0.78, 1.11)	0.91 (0.77, 1.08)	0.90 (0.76, 1.07)
Very safe				0.96 (0.79, 1.17)	0.91 (0.75, 1.12)	0.88 (0.72, 1.08)	0.88 (0.72, 1.07)
Neighborhood social cohesion					**1.07 (1.03, 1.11)**	**1.07 (1.03, 1.11)**	**1.07 (1.03, 1.11)**
Individual Stressors							
Perceived Stress						**0.95 (0.92, 0.97)**	**0.95 (0.92, 0.97)**
Discrimination							**0.80 (0.68, 0.95)**
