# Supplementary material for: Neighborhood Factors, Individual Stressors, and Cardiovascular Health Among Black and White Adults in the US: The Reasons for Geographic and Racial Differences in Stroke (REGARDS) Study
Source: JAMA Netw Open. 2023 Sep 29;6(9):e2336207. doi: 10.1001/jamanetworkopen.2023.36207 (PMC10543067; doi:10.1001/jamanetworkopen.2023.36207)
Supplement: Supplement 2. — Data Sharing Statement [file jamanetwopen-e2336207-s002.pdf]

Data Sharing Statement

Hines. Neighborhood Factors, Individual Stressors, and Cardiovascular Health Among Black and White Adults in the US. *JAMA Netw Open*. Published September 29, 2023. doi:10.1001/jamanetworkopen.2023.36207

Data
Data available: No

Additional Information
Explanation for why data not available: Data are available through request to the REGARDS study.
